# Identification of Two Novel Members of the Tentative Genus *Wukipolyomavirus* in Wild Rodents

**DOI:** 10.1371/journal.pone.0140916

**Published:** 2015-10-16

**Authors:** Juozas Nainys, Albertas Timinskas, Julia Schneider, Rainer G. Ulrich, Alma Gedvilaite

**Affiliations:** 1 Department of Eukaryote Genetic Engineering, Institute of Biotechnology, Vilnius University, Vilnius, Lithuania; 2 Institute for Novel and Emerging Infectious Diseases, Friedrich-Loeffler-Institut, Federal Research Institute for Animal Health, Greifswald-Insel Riems, Germany; University Hospital San Giovanni Battista di Torino, ITALY

## Abstract

Two novel polyomaviruses (PyVs) were identified in kidney and chest-cavity fluid samples of wild bank voles (*Myodes glareolus*) and common voles (*Microtus arvalis*) collected in Germany. All cloned and sequenced genomes had the typical PyV genome organization, including putative open reading frames for early regulatory proteins large T antigen and small T antigen on one strand and for structural late proteins (VP1, VP2 and VP3) on the other strand. Virus-like particles (VLPs) were generated by yeast expression of the VP1 protein of both PyVs. VLP-based ELISA and large T-antigen sequence-targeted polymerase-chain reaction investigations demonstrated signs of infection of these novel PyVs in about 42% of bank voles and 18% of common voles. In most cases only viral DNA, but not VP1-specific antibodies were detected. In additional animals exclusively VP1-specific antibodies, but no viral DNA was detected, indicative for virus clearance. Phylogenetic and clustering analysis including all known PyV genomes placed novel bank vole and common vole PyVs amongst members of the tentative *Wukipolymavirus* genus. The other known four rodent PyVs, Murine PyV and Hamster PyV, and Murine pneumotropic virus and Mastomys PyV belong to different phylogenetic clades, tentatively named *Orthopolyomavirus* I and *Orthopolyomavirus* II, respectively. In conclusion, the finding of novel vole-borne PyVs may suggest an evolutionary origin of ancient wukipolyomaviruses in rodents and may offer the possibility to develop a vole-based animal model for human wukipolyomaviruses.

## Introduction

The members of the *Polyomaviridae* family are small and non-enveloped DNA viruses. The circular dsDNA genome of polyomaviruses (PyVs) of approximately 5000 base-pairs (bp) is associated with host cell histone proteins H2A, H2B, H3 and H4 in a chromatin-like, supercoiled complex [1]. All PyVs possess a very similar genome organization. Five to nine proteins are encoded by early and late transcriptional regions separated by a non-coding control region (NCCR) which controls the transcription of both early and late promoters and contain the origin of DNA replication (ORI) [2, 3]. Early non-structural tumor (T) proteins are involved in the interference with cell cycle regulation and, in some cases, induction of cellular transformation or tumor formation [4, 5]. Late region usually encodes three structural proteins VP1, VP2 and VP3 which are translated from partially overlapping open reading frames (ORFs) and form icosahedral capsids of approximately 40–45 nm in diameter [3]. The VP1 protein is the major capsid protein which interacts with the cellular receptor molecule and is responsible for cellular entry [1]. Minor capsid proteins VP2 and VP3 may be necessary for specific encapsidation of the polyomavirus genome after genome replication [1]. Some of the PyVs encode additional regulatory and structural proteins known as VP4, agnoproteins or alternative large T ORF protein (ALTO) [6, 7]. The unique protein ALTO is encoded by several PyVs including Murine PyV (MPyV) and Hamster PyV (HaPyV) [7]. These additional proteins may act in virus release and/or genome replication, regulation of the cell cycle and DNA repair and/or capsid assembly or may even be incorporated into the viral capsid [6, 8–11].

Improvements in molecular diagnostic techniques led to identification of new polyomaviruses in different mammal, bird and fish species [12–17]. Because of growing quantity and variety of PyVs, the *Polyomaviridae* study group of the International Committee on Taxonomy of Viruses (ICTV) has recently suggested the division of the polyomaviruses into three tentative genera: *Orthopolyomavirus*, *Wukipolyomavirus* and *Avipolyomavirus* [3]. Based on the phylogenetic tree generated from an alignment of concatenated VP1, VP2 and LTag amino acid sequences, other authors have divided PyVs into five lineages: *Orthopolyomavirus I* and *II*, *Wukipolyomavirus*, *Avipolyomavirus* and *Malawipolyomavirus* [12]. Until now four PyVs were found in rodents. Rodent-associated MPyV and HaPyV belong to the tentative genus *Orthopolyomavirus I* [3, 18–20], whereas MPtV and Mastomys PyV (MasPyV) are members of the tentative genus *Orthopolyomavirus II*, [12, 16, 21]. Similar to MPtV, MasPyV was found to encode only two T antigens, large T antigen (LTag) and small T antigen (STag), whereas MPyV and HaPyV encode a third middle T antigen (MTag). Multimammate mouse (*Mastomys* spp.*)* and house mouse (*Mus musculus*), reservoir of MasPyV, MPyV and MPtV, belong to the family Muridae within the order Rodentia. In contrast to the Syrian hamster (*Mesocricetus auratus*) which is host of HaPyV and belong to subfamily Cricetinae, voles, like the bank vole (*Myodes glareolus)* and the common vole (*Microtus arvalis)*, represent another subfamily Arvicolinae of the rodent family Cricetidae. Both vole species show fluctuations in their population densities that result in high economic losses during peak densities in agriculture and/or forestry industry [22]. In addition, both species harbor important zoonotic pathogens, such as two different hantaviruses, cowpox virus and leptospires [23–25].

In this study, we report the identification, characterization and regional prevalence of two new wild rodent PyVs: bank vole polyomavirus (BVPyV) and common vole polyomavirus (CVPyV). Cloning, sequencing, ORF identification, phylogenetic and clustering analyses of the entire new BVPyV and CVPyV genomes let us to attribute these two new viruses to the *Wukipolyomavirus* genus.

## Methods

### Sample Collection

The animals were handled according to the national and European legislation, namely the EU council directive 86/609/EEC for the protection of animals. Collection of samples was carried out in accordance with institutional guidelines and permission of the national authorities. Bank and common voles were collected within hantavirus monitoring projects during 2011–2013 in four federal states of Germany [26] (Reil, Rosenfeld, Drewes, Schmidt, Turni, Wagner-Wiening, Jacob and Ulrich, unpublished data; see S1 and S2 Figs). Permits were issued by Regierungspräsidium Stuttgart; Landwirtschaft, Ländlicher Raum, Veterinär- und Lebensmittelwesen, Stuttgart (Baden-Wuerttemberg 35–9185.82/0261), by Landesamt für Natur, Umwelt und Verbraucherschutz Nordrhein-Westfalen, Recklinghausen (North Rhine-Westphalia 8.87–51.05.20.09.210), by Landesamt für Landwirtschaft, Lebensmittelsicherheit und Fischerei Mecklenburg-Vorpommern, Rostock (Mecklenburg-Western Pomerania 7221.3-030/09), and by Thüringer Landesamt für Lebensmittelsicherheit und Verbraucherschutz, Bad Langensalza (Thuringia 22-2684-04-15-107/09). Additional common voles were collected within the network “Rodent-borne pathogens” between 2004 and 2012 in seven federal states of Germany (Reil, Rosenfeld, Drewes, Schmidt, Turni, Wagner-Wiening, Schmidt and Ulrich, unpublished data) [27]. These animals were trapped and sacrificed by cooperation partners from forestry and consumer protection and food safety institutions during their official duties as part of their pest control measures. Therefore, specific animal ethics permits were not required.

After snap trapping or live trapping and subsequent cervical dislocation, animals were immediately frozen and stored at -20°C. Dissection and sample collection followed standard protocols as published recently [28]. Briefly, the frozen carcasses were thawed overnight and tissue samples taken during dissection in a defined order, starting with heart, then lung, liver, spleen, kidney, brain and intestine. For serological investigations, chest cavity fluid (CCF) was collected by washing the chest cavity with 1 ml phosphate-buffered saline. CCF and tissue samples were stored at -20°C until investigation.

Morphological species identification was verified by polymerase-chain reaction (PCR) amplification and sequencing of a partial cytochrome b gene, using DNA obtained from tail, heart or liver tissue following a previously published protocol [29].

### DNA extraction and amplification

Viral DNA was extracted from 200 μL of wild rodent CCF or kidney samples using GeneJET Viral DNA and RNA Purification Kit according to manufacturer’s recommendations (Thermo Fisher Scientific Baltics, Vilnius, Lithuania). Kidney samples were additionally treated for 1h with proteinase K (Thermo Fisher Scientific Baltics) before using GeneJET Viral DNA and RNA Purification Kit. Half of the sample of extracted viral DNA was used for PCR directly and other half of DNA was enriched and amplified by rolling circle amplification (RCA) as described previously [30, 31]. Briefly, 25 μL of viral DNA solution was treated with Plasmid-Safe ATP-Dependent DNase (Epicentre Biotechnologies, Madison, WI, USA). Then DNA was precipitated and amplified using phi29 DNA polymerase (Thermo Fisher Scientific Baltics) and exonuclease-resistant random primer (Thermo Fisher Scientific Baltics).

### PCR

Diagnostic PCR was carried out using DreamTaq Green DNA Polymerase (Thermo Fisher Scientific Baltics). The initial search of PyVs was performed according to a previously described nested PCR protocol and PyV primers T-1f, T-1r, T-2f, T-2r (see S1 Table) [32]. For the determination of the prevalence of the new PyVs two sets of primers were used: BVPyV-T-D3, BVPyV-T-R3, CVPyV-T-D3 and CVPyV-T-R3 (see S1 Table).

New complete PyV genome and VP1-encoding sequences were amplified using Phusion High-Fidelity DNA Polymerase (Thermo Fisher Scientific Baltics) and primers BVPyV-D1, BVPyV-R1, CVPyV-D1, CVPyV-R1, BVPyV-ATG, BVPyV-Stop, CVPyV-ATG and CVPyV-Stop (see S1 Table).

### Generation of plasmids and sequence determination

All DNA manipulations were performed according to standard procedures [33]. Enzymes and kits for DNA manipulations were purchased from Thermo Fisher Scientific Baltics. Recombinants were screened in *E*. *coli* DH10B cells. The PCR amplified PyV genomes and DNA fragments were cloned into pJET1.2 vector using CloneJET PCR Cloning Kit (Thermo Fisher Scientific Baltics) and sequenced using a BigDye Terminator v3.0 Cycle Sequencing kit on an ABI Prism 3130 Genetic Analyzer (Applied Biosystems Hitachi, Tokyo, Japan). Sequencing primers are listed in S1 Table. The PCR amplified, cloned and determined BVPyV and CVPyV partial or complete genome sequences were compared to the GenBank nonredundant nucleotide and protein databases using BLASTn and BLASTx, respectively.

The novel genome sequences were submitted to GenBank: BVPyV KS/14/281(GenBank: KR612368), BVPyV KS/14/289 (GenBank: KR612369), BVPyV KS/14/328 (GenBank: KR612370), BVPyV KS/14/336 (GenBank: KR612371), BVPyV KS/13/999 (GenBank: KR612372), CVPyV KS/13/947 (GenBank: KR612373), CVPyV KS/13/980 (GenBank: KR612374).

PCR-amplified BVPyV and CVPyV VP1-encoding sequences were cloned into previously described yeast expression vector pFX7 for target protein expression [34].

### Gene and protein annotation for the novel BVPyV and CVPyV genomes

Search for protein coding ORFs in PyV genomes was performed in two steps. First, all ORFs in the genome that begin at traditional initiation codons (AUG and GUG) were analyzed. Second, to investigate the presence of proteins that might be produced by alternative splicing of the early or late transcripts, putative exons of LTag, VP2 and VP1 genes were translated by all six open reading frames and full amino acid sequences were analyzed within non-redundant protein sequence database at NCBI by means of BLASTp [35]. ORF identification was based on splice donor/acceptor site analyses and alternatively similarity search with annotated proteins of other PyVs. First, possible splicing variants were analyzed by submitting LTag gene sequences to Human Splicing Finder database [36]. Then, splicing sites were evaluated in the context of protein sequences that they produce, and exons were identified based on homology to known PyV LTag proteins (S2 Table). ORI region was analyzed based on identified and defined SV40 and MCPyV ORI sequences [37–39].

### Expression, purification and characterization of recombinant VP1 proteins

The plasmids pFX7-BVPyV-VP1 or pFX7-CVPyV-VP1 were transformed into *Saccharomyces cerevisiae* strain AH22-214 (*a*, *leu2-3*,*112*, *his4-519*). Yeast transformants harboring plasmids with VP1 encoding genes were grown as described previously [40]. Yeast biomass harboring recombinant proteins was harvested by centrifugation and was stored at -20°C until use. The recombinant proteins were purified using a previously described protocol of ultracentrifugation in sucrose and cesium chloride gradients [40].

To prove formation of VP1-derived VLPs the samples of purified recombinant PyV-VP1 proteins were placed on 200-mesh carbon coated gold grids, negatively stained with 2% aqueous uranyl acetate solution and examined using Morgagni 268 electron microscope (FEI Inc., Hillsboro, OR, USA). The generation and characterization of Washington University PyV (WUPyV)-derived VP1 VLPs has been described recently [41].

### Indirect ELISA

The bank vole and common vole CCF samples were tested by an indirect ELISA. The microtiter plates with F-shape cavities (Nerbe Plus, Winsen/Luhe, Germany) were coated with 100 μL per well of recombinant BVPyV-, CVPyV- or WUPyV-derived VP1 VLPs and measles virus nucleoprotein [42] for negative control diluted in coating buffer (0.1 M sodium carbonate, pH 9.5) to a concentration of 2 μg mL^-1^ and incubated overnight at +37°C. The plates then were washed 4 times with washing buffer (10mM Tris, 30mM NaCl, 0.01% Tween-20, pH 7.2). After washing the coated plates were blocked by addition of 100 μL ROTI®-BLOCK (ROTH, Karlsruhe, Germany) per well and incubated for 1 h at room temperature (RT) on a plate shaker. Thereafter, the plates were rinsed four times with washing buffer. Wild rodent CCF samples were serially diluted (1:50, 1:100 and 1:200) with washing buffer containing 3% bovine serum albumin (BSA) and 100 μL of such diluted serum was added to each well. The polyclonal anti-WUPyV-VP1 mouse serum (raised against the WUPyV VP1 protein) was diluted 1:300 and used as positive control [41]. After incubation for 2 h at +37°C the plates were washed 8 times with washing buffer and incubated for 1 h with 100 μL/well of horseradish peroxidase (HRP)-conjugated goat anti-mouse IgG (BioRad, Hercules, CA, USA) diluted 1:600 in washing buffer. The plates were rinsed 5 times with washing buffer. HRP activity was detected by the addition of 100 μL of ready-to-use TMB Stabilized Chromogen substrate (Invitrogen, Camarillo, CA, USA) to each well. The plates were incubated for 30 min at RT in the dark, and the reaction was stopped by adding 150 μL of 10% sulphuric acid per well. The optical density (OD) was measured at 450 nm (reference filter 620 nm) in a microplate reader (Tecan, Groedig, Austria).

### Polyomavirus entire genome sequence data

Two strategies were implemented to extract all entire PyV genomes known to date (2015/27/04). First, 77 PyV RefSeq representative genomes as well as so called 'PyV neighbors' genome data according to the information on the internet site [43] were downloaded from National Center for Biotechnology Information (NCBI) repositories. Secondly, all NCBI nucleotide databases were screened for complete PyV genomes using data mining techniques. DNA sequences shorter than 3500 or longer than 8000 nucleotides (partial non-integrated or completely or partially integrated genomes) were skipped and remaining sequences were manually reviewed to confirm their PyV origin. At least one of T antigens and at least one of capsid proteins typical for PyVs (commonly LTag and VP1 were strongest representatives) were required to be present. The latter test was carried out using BLAST search for homologous proteins in NCBI protein database for candidate protein while expecting high extent of “polyomavirus” keyword for top ranked homologs found. If no “polyomavirus” protein was found using this method, a TBLASTN search was performed through the tested genome using known PyV proteins (LTag, STag, VP1, VP2) as bait. Results are described in detail in the PyV protein sequence data section below and S3 Table. Finally, both sets of genome sequences compiled by use of these two strategies mentioned above were combined and after removing duplicates it yielded an initial set of 1316 complete PyV genomes (including the 7 newly sequenced vole PyV genomes). Genome sequences of 15 PyVs had one (10 PyVs) or more (5 PyVs) ambiguous nucleotides that are not tolerated by some of the used methods. In order not to lose potentially unique genomes (by removing them from the dataset) the ambiguous nucleotides were replaced by most common regular nucleotides found at corresponding regions (identified by searching with a 41 nucleotide sequence centered on the ambiguous nucleotide) in other PyV genomes (all replacements and justifications can be viewed in S3 Table). Three genomes (namely JCPyV strain SA50329_06, and two HaPyV genome sequences: X02449 and PPCCGAAA), that had long unassigned nucleotide (N) stretches with no strong suggestions for replacement, were removed. Thereafter, the final set contained 1313 PyV genomes. Prior to viral DNA sequence clustering, a non-redundant genome sequence dataset was constructed as follows: genome-to-genome pairwise comparisons (described below) were used to build a set of corresponding DNA strands of PyV complete genomes and after that CD-HIT [44] was used to remove identical sequences. This resulted in a subset of 1029 PyV genomes (S3 Table). As a representative, but smaller PyV dataset was needed for phylogenetic analysis, CD-HIT was also used and sequences were filtered to 85% identity level. The used identity level of 85% is similar to genome sequence identity level (81–84%) which was proposed as criterion by the *Polyomaviridae* study group of ICTV to define separate PyV species [3]. NCBI RefSeq representatives were picked to represent CD-HIT clusters when available. After these manipulations a final set of 98 representative complete PyV genomes was obtained (S3 Table).

### Polyomavirus protein sequence data

FASTA libraries of protein sequences for LTag, STag, VP1 and VP2 were compiled using the two final sets of genomes (complete PyV genome sets of 1029 and 98 representatives). The fitness of selected representative sequences was further evaluated, according to each of the four proteins. Protein sequences of each of the 98 clusters were aligned using MAFFT [45] (L-INS-i algorithm) and resulting alignments were examined using JALVIEW [46, 47] to confirm the presence of typical cluster features in the selected representative sequences. Seven representative protein sequences were manually revised: missing C-terminal parts of protein (coded on separate exons) were added in case of two LTag proteins (MCPyV isolate MKL-1 and MCPyV isolate MCC339); the VP1 protein of Sea otter polyomavirus 1 isolate 6831–13 has not been previously annotated and was added to the set after TBLASTN search using VP1 sequences from other PyVs; the putative VP1 protein of bat polyomavirus isolate BtMp-PyV/SX2013 was extended at the N-terminus to fit better to other VP1 proteins. Full details on protein representative sequence rearrangements are given in S3 Table. We failed to identify STag sequences in black sea bass and guitarfish PyV genomes, despite having used TBLASTN and PSI-TBLASTN methods to scan corresponding genomes with all other representative STags and constructed STag profiles as baits, respectively. STag profiles were built with PSI-BLAST [48] using non-redundant protein set (further filtered to 80% sequence identity with CD-HIT) obtained from NCBI. Obtained representative protein datasets were used for phylogenetic analysis.

### Multiple sequence alignments for phylogenetic analysis

Multiple sequence alignments for the four protein datasets were produced using MAFFT (L-INS-i algorithm). By means of PSI-BLAST [48], these alignments were used to find close relatives of the analyzed proteins within non-redundant protein sequence database at NCBI as well as homologous proteins with known 3-dimensional (3D) structure to be used for alignment refinement. For each of the four sets of proteins, obtained sequences were aligned using L-INS-i (two versions: with “-ep 0.123” parameter (which allow higher restrictions for gap extension as recommended at [49]) or without [default]) and PROMALS3D [50–51]. PDB structures used for LTag: 4gdfA and 3pf4 (represents SV40),4bnpA (JCPyV), 3qfqA(MCPyV); for STag: 2pf4E (SV40), 1fafA (MPyV); for VP1: 1sieA (MPyV), 4mbyA (B-lymphotropic PyV), 4fmh (MCPyV), 4posA (HPyV9,), 4mj0A (BKPyV), 3bwrA (SV40), 4x16 (JCPyV), 1cn3A (MPyV strain Crawford), 4pcgA (HPyV6), 4pchA (HPyV7), 3s7x (WUPyV), 3s7vA (KIPyV); for VP2: 1cn3F (structural data for 29 aa fragment VP2 of MPyV, small plaque variant of strain Crawford). Refined final alignments of reference proteins (n = 98) were obtained after removal of the extra sequences from alignments. Resulting alignments were concatenated into alignments of all four analyzed proteins. To avoid duplication of information, N-terminal part (~70 amino acid-long fragment) identical between LTag and STag was removed in alignment for STag before concatenation. A filtered version of the alignment for phylogenetic analysis was obtained by removing weak alignment positions from each of the four alignments with GBLOCKS [52–53] using mildest filtering parameters on GBLOCKS server [54].

### Phylogenetic analysis

Phylogenetic analysis was carried out using maximum likelihood (ML) algorithm. For each alignment, ProtTest [55] was used to select the best-fit evolutionary model to be used in phylogenetic analysis (excluding estimate of proportion of invariable sites (I) because of comparatively small test set). Le and Gascuel [56] amino acid substitution matrix (LG) with GAMMA (G) model of rate heterogeneity and optimization of substitution rates (F) was found as best model (LG+G+F) for all four proteins and, thus, this model was used in final analysis with concatenated alignments. For every analysis we produced 500 trees and 1000 bootstrap replicates using RAxML [57]. Convergence test as implemented in RAxML was used to confirm that obtained number of bootstrap replicates [58, 59] was sufficient (in all cases 50–150 replicates were sufficient). Phylogenetic trees were visualized using DENDROSCOPE [59]. A majority rule consensus tree of all 500 trees was made to evaluate their differences. The leave-one-out approach for assessing site-specific congruence/incongruence with the underlying tree based on the evolutionary placement algorithm [60] was used as implemented in RAxML. Several methods were used to evaluate the confidence of branch placements within the tree. In addition to the standard bootstrapping method, calculated Shimodaira–Hasegawa (SH) [61], Internode Certainty (IC) and Tree Certainty (TC) values [62, 63] were calculated. Uncertain taxa within the constructed phylogenetic tree were also identified alternatively by use of RogueNaRok [64].

### Comparison of entire PyV genomes

To compare whole genome sequences, a method that does not require sequence alignments to get a final estimate of similarity was developed. The implemented sequence comparison methodology is very simple and some similarity might be found to procedures used by Fitch [65, 66]. Analysis was done by gathering statistics of best pairs of sequence spans of the length “w” taken from two genomes under comparison. Thus, all w-mers taken from one genome were compared with w-mers from a second genome by means of a sliding-window procedure and counted only pairs of w-mers which had higher count of identical nucleotides in their alignment than some predefined critical value (in this work w = 50 and critical value n = 30). The critical value “n” was determined by modeling statistics of comparison results for randomized genome sequences. The value was set to mean low level of random background (no higher than 10–15% of meaningless hits in comparison to lowest similarities between PyV genomes).

We suggest that the probability p of total count of pairs of w-mers of at least S to appear by chance during genome comparison could be calculated using the extreme value distribution [67–69]:

$$p\left(S\geq x\right)=1-\text{exp}\left(-e^{-\lambda \left(x-u\right)}\right)$$ (1)

The equation has two parameters: the characteristic value *u* can be thought of as the mean of x, and λ is a scale parameter. Best fit values for u and λ for gathered statistics of randomized genomes were 18 and 0.1 respectively (for case of w = 50 and n = 30 used in this study).

### Cluster analysis of genome sequences

To cluster polyomavirus genomes, precalculated probabilities p (Eq 1) for all 1029x1029 polyomavirus genome pairs were used as input for CLANS [70]. The genomes were clustered using different levels of p to highlight different evolutionary relationships. Initially dynamic clustering was run using 3D space, but final representations of clusters were transformed into 2D space.

## Results and Discussion

### Identification and genome analysis of new PyVs

Application of the nested broad-spectrum PCR technique [32], resulted in the detection of approximately 250 bp-long PCR products for six out of 172 bank voles and nine out of 85 common voles (S4 Table). After DNA sequence determination, a BLASTn search in GenBank revealed two novel PyV-related nucleotide sequences with the highest similarity of 75% and 74% to the LTag-encoding region of WUPyV and MCPyV respectively. The sequence similarity of both 250 bp DNA fragments to other members of the tentative genus *Wukipolyomavirus* was not significant. The similarity of the bank vole-derived DNA sequence to other rodent PyVs was not significant, but the DNA sequence amplified from common voles was 69% identical to HaPyV LTag sequence. The entire PyV genomes from five bank voles and two common voles were amplified, cloned and sequenced (S4 Table).

Three of five BVPyV genomes amplified from CCF samples of bank voles (KS/14/281, KS/14/289, KS/14/328) captured at the same location (Crailsheim, Baden-Wuerttemberg; see S1 Fig, site #3) were 5032 bp long and differed by one to five single nucleotide polymorphisms (SNP) from rodent KS/14/336-derived, 5031 bp-long BVPyV genome (S2 Table). The fifth genome amplified from bank vole sample KS/13/999 captured at another location (Gotha, Thuringia, see S1 Fig, site #2) one year earlier was 5011-bp long and 98% identical to the genome of BVPyV-KS/14/281. The 81 SNPs in BVPyV KS/13/999 genome compared to BVPyV KS/14/281 strain were mainly located in intergenic regions, VP1 gene and C-terminal end of LTag encoding gene. The remaining part of early region encoding LTag and STag antigens (2300 bp genome fragment) contained no mutations at all. A variability of viral strains from the same host was demonstrated for most of human and non-human PyVs [71–74]. However, sequenced CPyV genomes from common voles collected in distant regions of Germany demonstrated much less divergence than genomes of BVPyV detected in bank voles. Thus, the CVPyV genome sequence generated from CCF sample of common vole KS/13/947 captured in Gotha, Thuringia (see S2 Fig, site #9) differed from CVPyV genome generated from kidney sample of rodent KS/13/980 (Jeeser, Mecklenburg-Western Pomerania, see S2 Fig, site #3) by one SNP only. Both CVPyV genome sequences were 5024 bp long (S2 Table).

**Fig 1:**
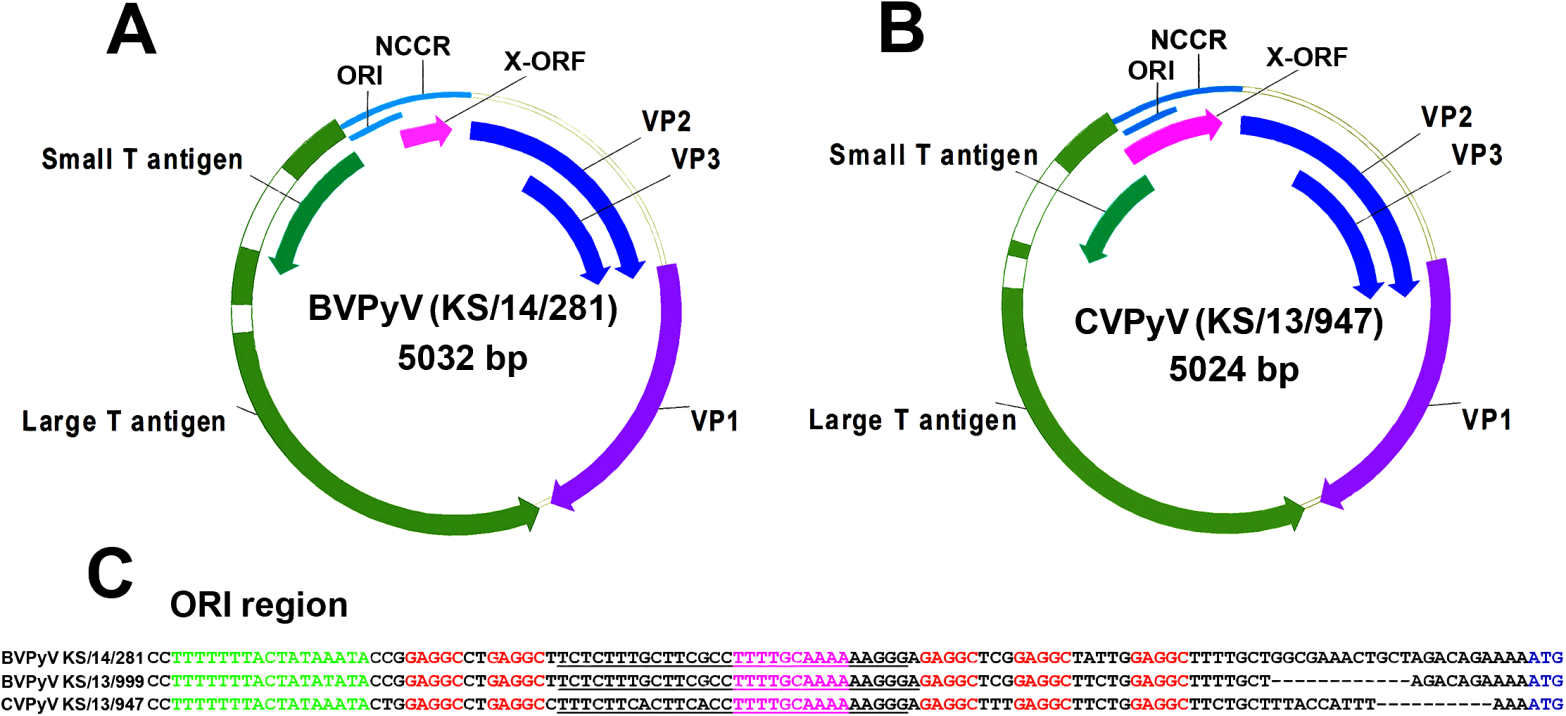
Schematic presentation of the genome organization of BVPyV (A) and CVPyV (B) and alignment of ORI region sequences of BVPyV strains (KS/14/281 and KS/13/999) and CVPyV strain KS/13/947 (C). The positions of LTag introns are shown uncolored. Putative LTag binding sites (GAGGC pentanucleotides) are shown in red; AT-rich region/TATA box is shown in green; palindromic repeats shown in magenta; early palindrome (EP) region is underlined; LTag ATG codon is shown in blue (according [38, 39]).

All seven cloned and sequenced genomes had the typical PyV genome organization, including putative ORFs for early regulatory proteins (LTag and STag) on one strand and for structural late proteins (VP1, VP2 and VP3) on the other strand (Fig 1A and 1B). Both regions were separated by the NCCR. LTag and STag encoding sequences were identified based on homology to known PyV proteins, on genomic location and sequences resembling classical eukaryotic splice donor and acceptor consensus signals. Two splicing sites were detected for LTag early transcript (S2 Table). Such analysis showed that LTag mRNA contains three exons encoding 651 aa protein for four BVPyV strains (or 650 amino acids, aa, for KS/13/999) and 642 aa for both CVPyV strains. Further, the early genome regions of CVPyV and BVPyV do not encode MTag or ALTO proteins, which is in contrast to HaPyV or MPyV [7]. However, further experiments with mRNA isolated from virus infected cells are needed to fully investigate the presence of LTag splicing variants because LTag in other rodent PyVs are encoded by only two exons. An additional putative ORF (tentatively named X-ORF) was found in the NCCR region of BVPyV and CVPyV (Fig 1A and 1B, magenta arrow). ORF sizes and locations were not conserved between BVPyV strains KS/14/281 (162 bp) and KS/13/999 (189 bp) or CVPyV (426 bp) genomes (Fig 1 and S2 Table). The putative proteins encoded by these X-ORFs in BVPyV strains KS/14/281 (53 aa) and KS/13/999 (62 aa) or both CVPyV strains (141 aa) had different length, were less than 35% identical in their amino acid sequences and had no sequence similarity to any protein from protein data bases, so they are unlikely to be synthesized and/or of functional relevance.

The genome alignments to the nucleotide sequences in GenBank showed that the closest known relative was WUPyV which in alignable regions on a whole genome basis shared 79% identity with BVPyV and 67% identity with CVPyV. BVPyV and CVPyV DNA sequence identity over the whole genome (alignable region did not include part of NCCR) was 83%, so according to the criteria of the *Polyomaviridae* study group of ICTV [3] these two viruses may represent two new separate PyV species.

The difference of the genome length of BVPyV strains KS/13/999 (5011 bp) and KS/14/281 (5032 bp) are due to indels within the NCCR sequence and intergenic region between LTag and VP1 genes, except deletion of one codon encoding N649 in LTag sequence and one nucleotide in the first intron of LTag. The insertion of four nucleotides in noncoding region between LTag and VP1 genes in BVPyV (KS/13/999) genome was also determined. This is in line with previous studies indicating that the highest level of variation is common for noncoding regions of PyVs [73, 75].

NCCR sequences of PyVs are suggested to be important for the host adaptation of certain PyVs [75]. The core region of both novel vole-derived PyVs demonstrated a high level of sequence conservation. Five putative LTag binding sites (GAGGC), palindromic repeats and early palindrome (EP) region [38, 39] were detected in NCCR ORI region of both viruses (Fig 1C). Interestingly, a 12 bp deletion in the NCCR region of BVPyV variant KS/13/999 did not change the arrangement of main ORI elements found in KS/14/281 and other three BVPyV variants. This deletion had changed only the distance between ORI and LTAg ATG codon, placing it closer to ATG (Fig 1C). Analogously, ORI region sequences in CVPyV and BVPyV genomes were very similar with the same arrangement and sequences of ORI elements but CVPyV ORI was positioned closer to LTag ATG codon alike to BVPyV variant KS/13/999 (Fig 1C). In contrast, the second part of NCCR sequences, where a variety of cellular transcription factors bind and regulate the NCCR, were more diverse and only 65% identical between BVPyV and CVPyV or 95% between BVPyV variants KS/14/281 and KS/13/999.

### Generation of BVPyV- and CVPyV-derived VP1 VLP

Full-length comparison of the VP1 gene sequences revealed that KS/13/999 strain sequence differed from other BVPyV VP1 sequences by 31 SNPs, but only one SNP was non-synonymous (A327T). For this reason one gene was amplified from strain KS/14/281 and used for the expression of BVPyV-derived VP1 in yeast. CVPyV-derived VP1 gene was amplified from strain KS/13/947.

**Fig 2:**
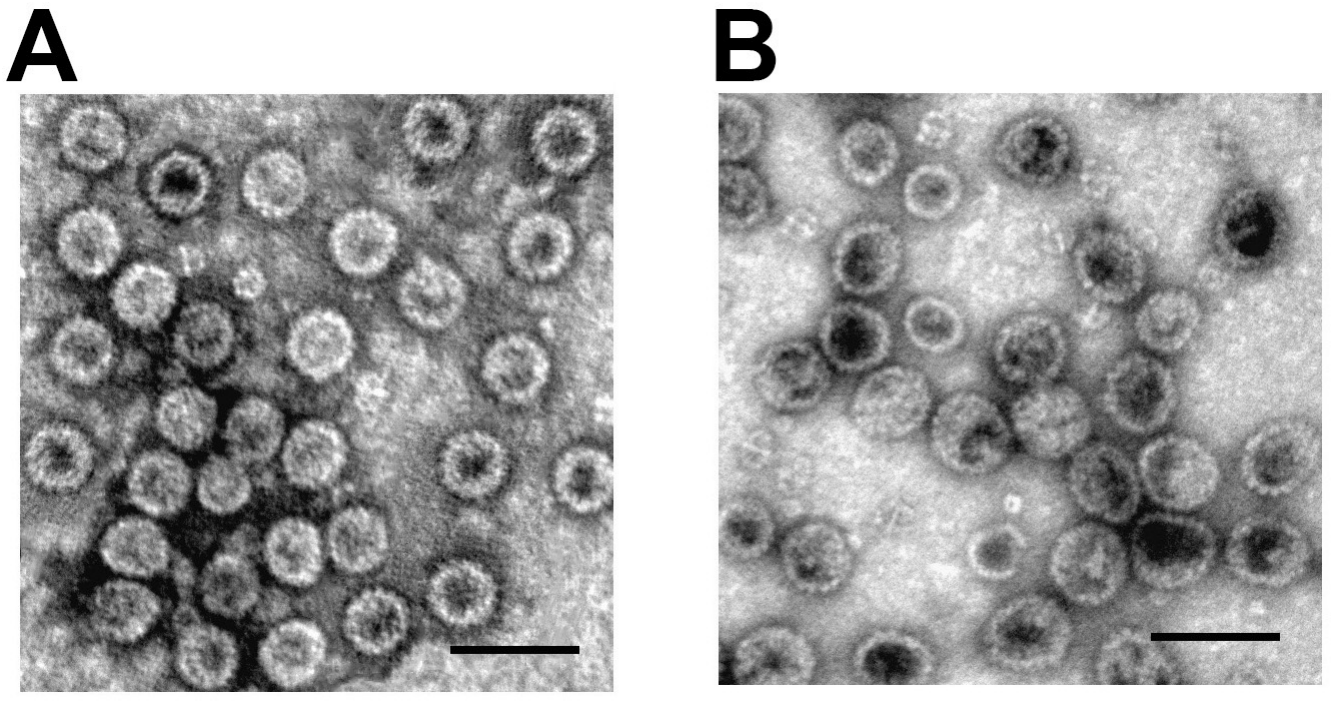
Detection of BVPyV (A) and CVPyV (B) VP1 protein VLP formation by electron microscopy. Bars, 100 nm.

Recombinant VP1 proteins of BVPyV and CVPyV were efficiently generated in yeast. The ability of the VP1proteins to self-assemble into VLPs was tested after purification by electron microscopy (EM) analysis. It was demonstrated that VP1 proteins derived from both new viruses were assembling into PyV-like VLPs (Fig 2A and 2B). The size of BVPyV- derived VLPs was 45–50 nm and that of CVPyV-derived VLPs 50–55 nm in diameter. The VP1 VLPs were used for serological screening of vole CCF samples by indirect ELISA. A parallel analysis of WUPyV-VP1-derived VLPs should allow conclusions on the potential cross-reactivity of antibodies with VLPs of this PyV and the two novel vole PyVs.

### Prevalence and host specificity of vole-associated PyVs

Bank vole and common vole kidney- and CCF-derived and RCA-enriched DNA samples were examined using specific diagnostic primers for both viruses (S1 Table). The novel assay detected BVPyV in 23 CCF and 41 kidney samples of bank voles (S4 Table). Samples of CCF and kidney tissues were both positive for BVPyV only in two animals. All other samples were PCR-positive only in one of two examined tissues. BVPyV VP1-VLP-based indirect ELISA detected 17 CCF samples with anti-BVPyV VP1 antibodies, with only 6 of them being identified by PCR as positive. In most cases, BVPyV VP1 VLP-specific antibodies were also recognized by WUPyV VP1 VLPs (16 from 18), but less frequently by CVPyV VP1 VLPs (7 from 18). Taken together, 73 of 172 (42.4%) bank voles showed signs of previous or acute BVPyV infection (S4 Table). The positive animals originated from nine out of ten trapping sites from the three investigated federal states Baden-Wuerttemberg, Thuringia and Mecklenburg-Western Pomerania, indicating a broad geographical distribution of the virus. Only at one site in Baden-Wuerttemberg (Steinheim) none of the two bank vole samples was positive (S4 Table).

Diagnostic CVPyV-specific PCR identified 11 of 85 animals to be CVPyV positive in one of the tissues used for PCR (S4 Table). ELISA analysis of available CCF samples of common voles showed that only five of 72 rodents had antibodies against CVPyV VP1 VLPs (S4 Table). These antibodies efficiently recognized WUPyV VP1 (in all five animals) and less efficiently BVPyV VP1 VLPs (two of five animals). Antibodies were mainly found in PCR-negative animals. Taken together, 15 of 85 (17.6%) common voles indicated signs of a previous or ongoing CVPyV infection. Positive voles originated from seven states of Germany (S4 Table). Infected animals were found in all eight federal states investigated.

Our results demonstrated that about 42% of bank voles and 18% of common voles were infected with new PyVs. Both viruses were found in CCF and kidney samples. Interestingly, despite a 90% amino acid sequence similarity of BVPyV and CVPyV VP1 proteins antibodies from rodents positive with VLPs of these new viruses were cross-reacting with VP1 VLPs of each other less efficiently, than with WUPyV VP1 which is only 48% identical to VP1 proteins of both new PyVs (S4 Table). The exclusive detection of viral DNA or of anti-VP1 antibodies in bank and common voles might be in contrast to the usually observed establishment of a chronic PyV infection in their hosts [1]. PyV persistence might be established in the kidney with virus release or alternatively PyVs might stay in a latent state [1]. In line, antibodies against virus proteins, especially VP1, are detected soon after infection and remain for a long time, while virus DNA might be not detected anymore [1, 76]. Future studies would have to prove a latent state of the infection with the novel PyVs resulting in a failure of virus DNA detection in anti-VP1-positive animals.

### Phylogenetic analysis

**Fig 3:**
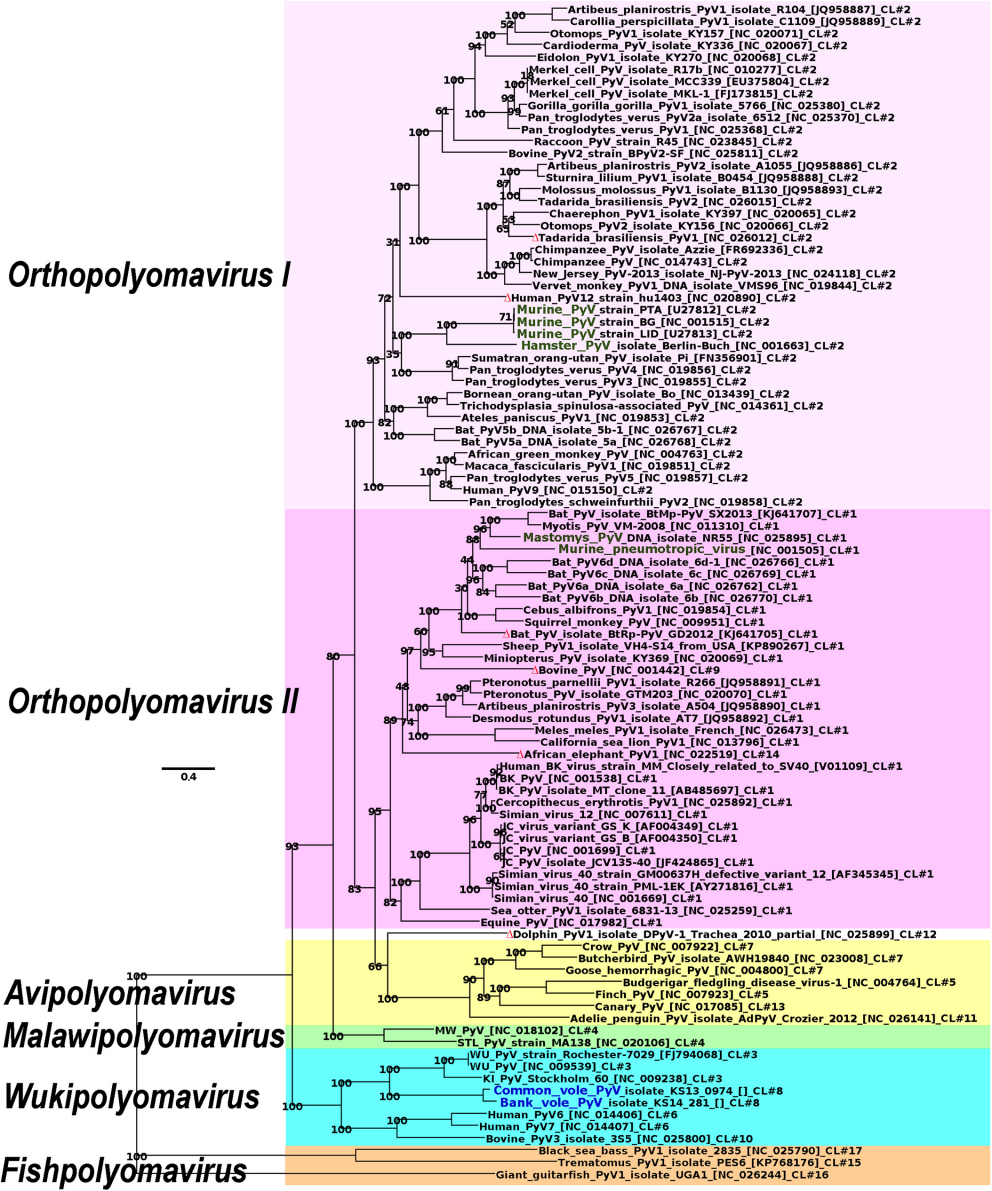
Phylogenetic tree of 98 representatives of *Polyomaviridae* family obtained using ML methods for concatenated LTag, STag, VP1 and VP2 protein sequence alignment. The tree is rooted at the most distant PyVs, namely fish polyomaviruses. Numbers at the nodes indicate statistical support (bootstrap values) for branch placements. Red triangles mark taxons with least confidence in their placement, as determined using RogueNaRok. The five lineages: *Orthopolyomavirus I*, *Orthopolyomavirus II*, *Avipolyomavirus*, *Malawipolyomavirus* and *Wukipolyomavirus* are highlighted in different colors. Rodent polyomaviruses Murine PyV (MPyV), Hamster PyV (HaPyV), Murine pneumotropic virus (MPtV) and Mastomys PyV (MasPyV) are labeled in green. The novel viruses BVPyV and CVPyV are labeled in blue.

LTag, STag, VP1 and VP2 proteins known to be common for all PyVs were selected for phylogenetic analysis. As the quality of phylogenetic analysis depends highly on the quality of provided sequence alignments, in this study four separate alignments for each LTag, STag, VP1 and VP2 proteins as well as a concatenated one of 98 representative PyVs were produced using three different methods. A maximum likelihood tree with highest bootstrap support values and lowest uncertainty of branch placement was obtained for MAFFT alignment with “—ep 0.123” when the alignment was not reduced by use of GBLOCKS (Fig 3).

Most uncertainly placed taxa pointed out by RogueNaRok server (reviewing taxa support values below 80%) were HPyV12 (0.910), African elephant PyV (AePyV; 0.099), bovine PyV (BoPyV; 0.091) and dolphin PyV (DoPyV; 0.035). Lower uncertainty values were shown for three PyVs, bat isolate BtRp (0.013), Tadarida brasiliensis 1 (0.004) and JCV135-40 (0.002).

The three tentative genera (*Orthopolyomavirus*, *Wukipolyomavirus*, and *Avipolyomavirus)* [3] can be easily recognized as distinct clades in our tree. In line with a previous study [12], the position of *Avipolyomavirus* group separates the *Orthopolyomavirus* group into two subgroups: *Orthopolyomavirus I* and *Orthopolyomavirus II*. Additionally besides the four above mentioned groups, Feltkamp et al. [12] recommended the distinction of one more lineage–*Malawipolyomavirus* (occupied by MWPyV, STLPyV, MXPyV, and HPyV10) which is also grouped together in our phylogenetic analysis (Fig 3). Recently identified three fish PyVs (black sea bass [77], giant guitarfish (RefSeq NC_026244; unpublished) and *Trematomus* (RefSeq NC_026944; unpublished) form a sixth group.

New bank and common vole PyVs are confidently incorporated into the *Wukipolyomavirus* group of PyVs (bootstrap support values of 100) forming a vole/WUPyV/KIPyV sister clade to the HPyV6/HPyV7/BPyV3 clade. Interestingly, in discussing of possible evolutionary pathways of WU/KI PyVs, Gaynor and colleagues proposed that these PyVs might have originated from rodent (murine) PyVs [78]. Their proposal was based on the lack of a C-terminal “host range” domain in LTag and agnoprotein. Thus, our findings of new vole PyVs within *Wukipolyomavirus* group support their proposal. The other rodent PyVs were found at different positions within the tree showing stronger similarities for MPyV and HaPyV on one hand and MPtV and MasPyV on the other (Fig 3).

### Whole genome comparisons

**Fig 4:**
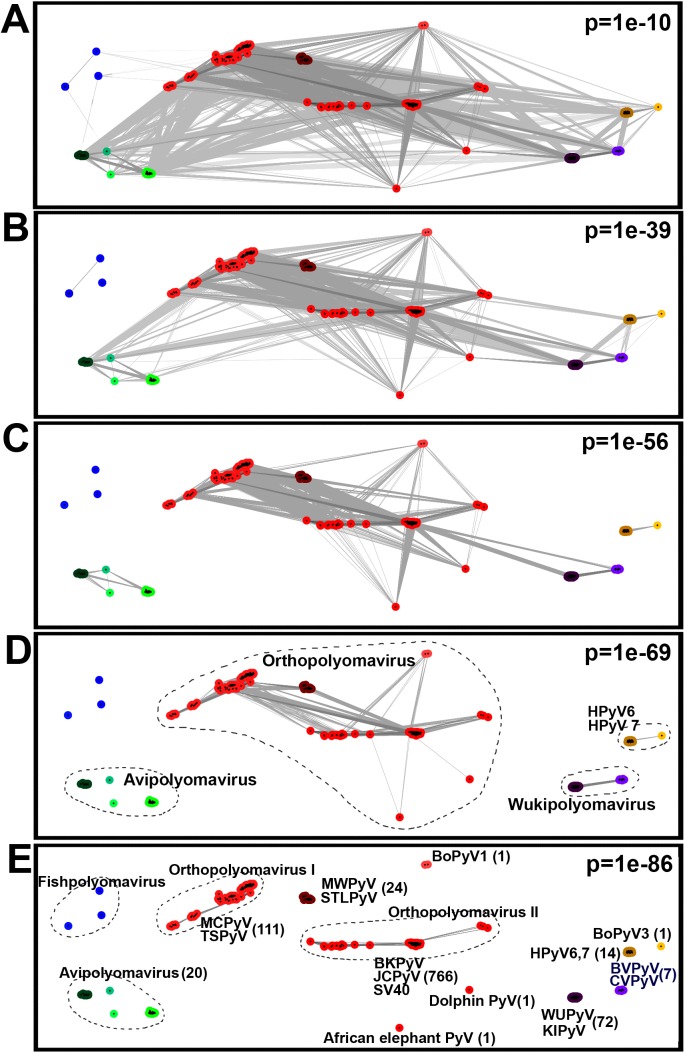
Relationships between PyV genomes, as shown at various cutoff similarity values. **(**A) cutoff p = 1x10^-10^: all PyVs are nested indicating a common nature of all PyVs (not excluding fishpolyomaviruses); (B) cutoff p = 1x10^-39^: all PyVs are nested, except fishpolyomaviruses; (C) cutoff p = 1x10^-56^: Avipolyomaviruses are separated; (D) cutoff p = 1x10^-69^: Wukipolyomaviruses are separated; (E) cutoff p = 1x10^-86^: Orthopolyomaviruses are divided into 3 main lineages—*Orthopolyomavirus I*, *Orthopolyomavirus II* and *Malawipolyomavirus*. To note, 1x10^-86^ level indicates closest relationships, while 1x10^-10^—most distant. Grey lines connect pairs of similar PyV genomes at selected cutoff level of p.

To evaluate the relatedness of PyVs, an alternative algorithm was developed in this study. Novel (global) measure for similarity of genome sequences was introduced by counting pairs of short fragments (of length w) that share higher sequence identity than some predefined value (n identical nucleotides per w) when aligned. This simple measure empowered comparison of any potentially meaningful w-mer pairs, including genomic stretches coding conservative functional domains of proteins, specific regulatory regions of genome, microRNAs or any other important signals within PyVs DNA sequence, without the use of any additional information. Sequence dynamic clustering software CLANS, with the calculated pairwise similarity scores (p) of PyV genomes as input, was used to investigate relatedness between all analyzed genomes (Fig 4).

At p = 1x10^-69^ all orthopolyomaviruses formed a homogeneous cluster, while other groups of PyVs were spread into smaller clusters or had orphans not showing relatedness at this level of similarity (Fig 4D). Thus, wukipolyomaviruses were separated into two clusters: one cluster consisted of WUPyVs, KIPyVs and new PyVs from bank and common voles and other was composed of HPyV6, HPyV7 and BoPyV3 (Fig 4D). The *Avipolyomavirus* group was separated into four subgroups while fish PyVs did not indicate any connections even at p = 1x10^-56^ similarity level (Fig 4C). Similarity level p = 1x10^-86^ would fit sufficiently with scheme of classification of PyVs where *Orthopolyomavirus* group is split into at least three lineages suggested by others: *Orthopolyomavirus I*, *Orthopolyomavirus II* and *Malawipolyomavirus* [12]. Low similarity of the three PyVs AePyV, DoPyV and BoPyV1, to other orthopolyomaviruses could indicate loss of some group specific features in genome sequence. It is interesting to note that during the phylogenetic analysis these three PyVs were also pointed out by RogueNaRok as having uncertain placement.

Inside *Wukipolyomavirus* cluster at p = 1x10^-86^ genome similarity level, human PyVs and non-human PyVs separate into distinct clusters (BVPyV/CVPyVs–from WU/KI PyVs separates at p = 1x10^-83^, Bovine PyV3 from HPyV6/HPyV7 separates at p = 1x10^-73^). Despite this, it should be mentioned that most close representatives of HPyVs 6 and 7 are WU/KI PyVs (at p = 1x10^-45^ multiple connections) and so these HPyVs could be assigned into one cluster. A more detailed picture of the relatedness levels of PyVs is given in a colored matrix form in S5 Table.

**Fig 5:**
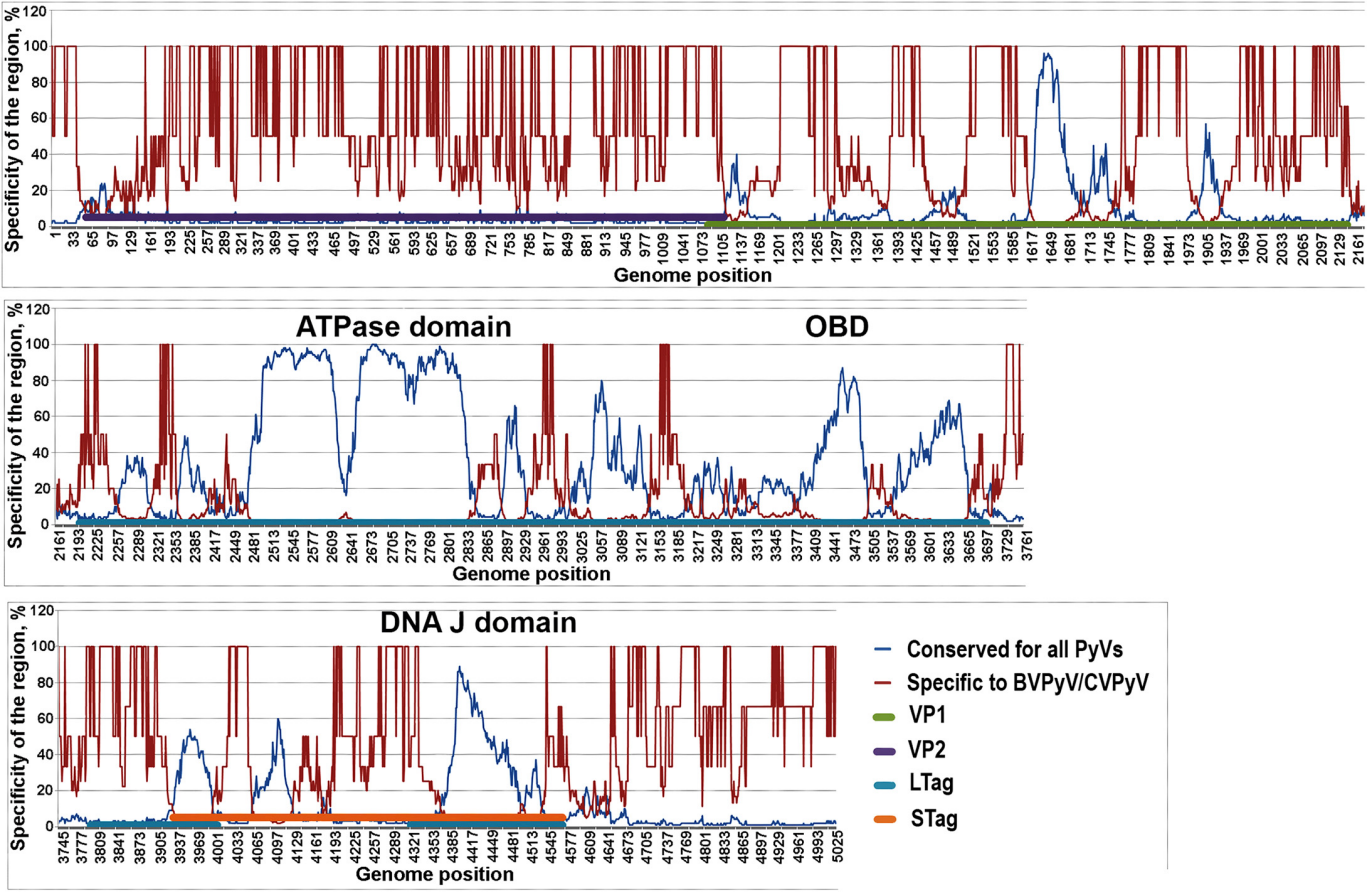
Sliding window analysis of genome sequences specific to CVPyV and BVPyV compared to other PyVs (%). The short fragments of a length w = 50 nt of BVPyV and CVPyV genomes were compared with w-mers of all PyV genomes by means of a sliding-window procedure. Only pairs of w-mers which had higher count of identical nucleotides in their alignment than some predefined critical value n = 30 when aligned were determined as similar. VP1, VP2, VP3, LTag, and STag genes are labeled with different colors. The nucleotide positions in genome are indicated at the bottom.

Additional investigation of whole genome comparisons were also performed by analyzing PyV genomes for the DNA sequence features conserved between all PyVs and highlighting sequence features that are specific for CVPyV and BVPyV (Fig 5). The VP2 gene sequence carries little similarity among all PyVs (including WUPyV) except for a short region encoding the N-terminal end where a myristoylation target sequence is located. However, this gene of both vole PyVs is highly similar (88% identical). There could be several explanations: a recent branching event in the evolutionary history and/or presence of conserved specific VP2 function of vole PyVs. VP1 gene contains a few conserved sequence features (overall identity of BVPyV and CVPyV VP1 gene sequences is 82%). Notably the highest similarity between the VP1-coding sequences of all PyVs is seen in a region in the middle of the gene that encodes part of the core β-barrel structure. While both of the late region genes show little similarity among all PyVs, the LTag gene sequence is much more conserved. Conserved sequence features can be linked with known domain locations. All three main domains: ATPase, DnaJ domains and origin binding domain (OBD) retain similarity between all PyVs and the new ones as well (Fig 5) [79]. The largest conserved 350 bp length region encode Walker A and B boxes as well as the Surface Loop motif of the AAA+ ATPase domain [79]. Thus, this additional analysis on whole genome comparison algorithm fully confirms the current knowledge of the PyV genome regions and conservation of highly similar domains and motifs and strengthens the results of the CLANS analyses.

## Conclusions

The growing number of newly discovered PyV uncovered the polyomavirus diversity which is reflected in phylogenetic tree as well as in CLANS analysis. The true genomic variety of PyVs is still unknown as well as the true host range or evolutionary rates. The numerous distinct PyV species found in humans or non-human primates seems in contrast with only four PyVs species known in rodents (MPyV, HaPyV, MPtV, and MasPyV). Rodents represent the largest order of mammalian species (contain 40% of all mammal species) and are a reservoir of more than 60 known zoonotic human pathogens [80, 81]. All four until now known rodent PyV genomes consistently grouped in *Orthopolyomavirus* I and II genera. Phylogenetic analysis and whole genome comparison analyses were used to classify the new bank vole and common vole PyVs as members of the tentative *Wukipolymavirus* genus. The finding of these novel PyVs suggest that more PyVs belonging to other clades might be present in other rodents and awaits their future discovery. The identification of the vole PyVs might help better understand the true genetic diversity and evolutionary history of the wukipolyomaviruses. The sequence divergence and phylogenetic positions of HaPyV and the vole-associated novel viruses may indicate a murine origin of HaPyV, instead a cricetide origin. In addition, the currently known diversity of PyVs cannot be explained by a long-term co-evolution of PyVs and mammalian hosts, but rather a split of an ancestral PyV into different lineages that thereafter might follow a co-evolution mechanism. Further, the finding of these vole PyVs may suggest that the wukipolyomavirus ancestor might have existed in a rodent-like animal. In addition, the vole wukipolyomaviruses may allow the development of novel rodent models for detailed studies on transmission and pathogenicity of the corresponding human PyVs.

## Supporting Information

S1 Fig Trapping sites of bank voles (*Myodes glareolus*) in Germany. The map was generated using the ArcGis program package. Source: Geobasis-DE/BKG/GeoNutzV.
(TIF)

S2 Fig Trapping sites of common voles (*Microtus arvalis*) in Germany. The map was generated using the ArcGis program package. Source: Geobasis-DE/BKG/GeoNutzV.
(TIF)

S1 Table Primers used to detect, amplify and characterize BVPyV and CVPyV genomes. (PDF)

S2 Table Vole trapping site information and genome characterization of BVPyV and CVPyV. (PDF)

S3 Table PyV genome and protein sequence datasets used in this study. (XLSX)

S4 Table Results of PCR and ELISA analysis of bank vole and common vole CCF and kidney samples. (XLSX)

S5 Table Colored matrix form of levels of PyVs relatedness generated by counting pairs of short fragments of a length w = 50 nt that share higher sequence identity than predefined value n = 30 nt when aligned. (XLSX)
