# Supplementary figures and images for: Identification of Two Novel Members of the Tentative Genus Wukipolyomavirus in Wild Rodents
Source: PLoS One. 2015 Oct 16;10(10):e0140916. doi: 10.1371/journal.pone.0140916 (PMC4608572; doi:10.1371/journal.pone.0140916)

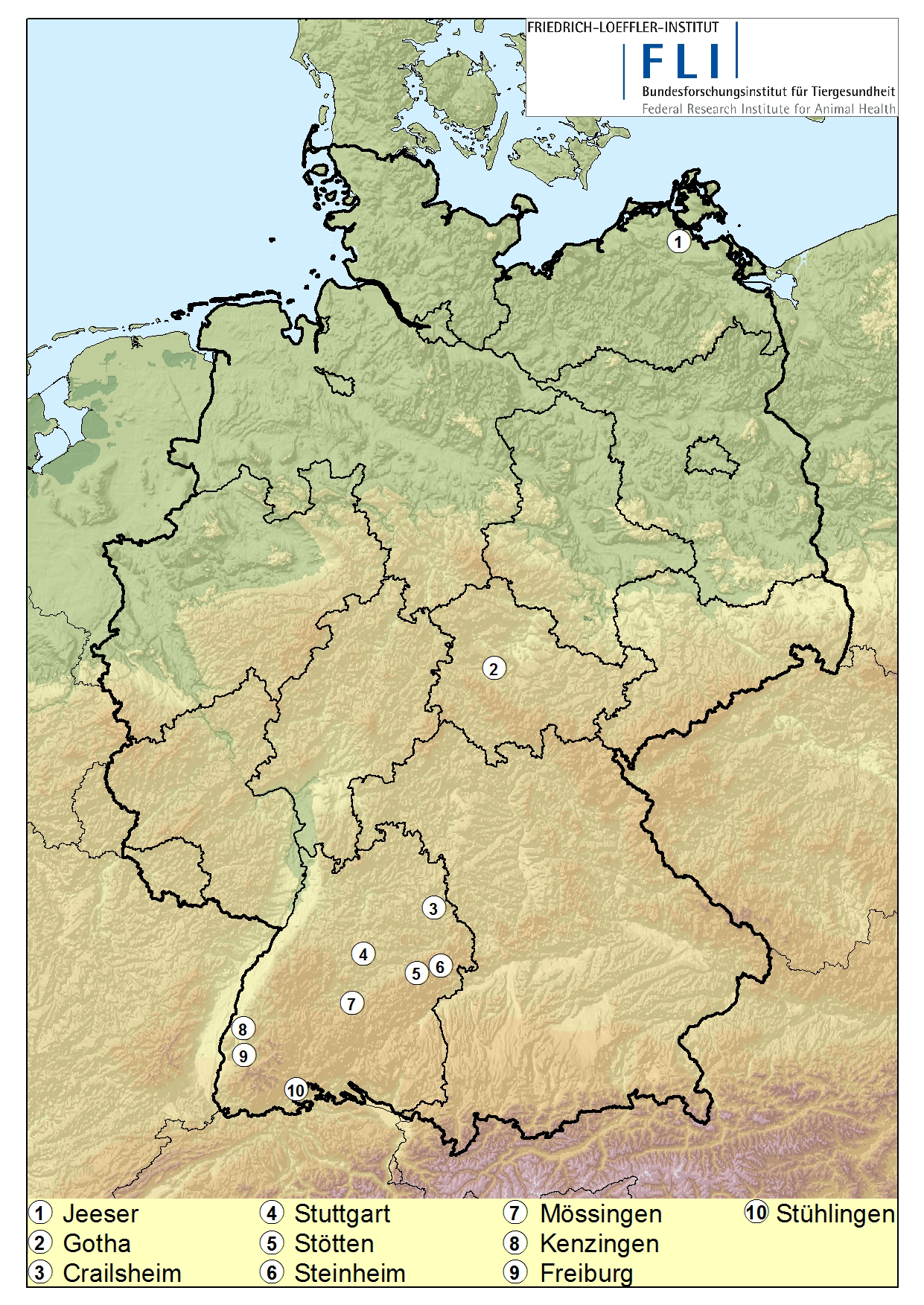

Supplement: S1 Fig — The map was generated using the ArcGis program package. Source: Geobasis-DE/BKG/GeoNutzV. (TIF) [file pone.0140916.s001.tif]

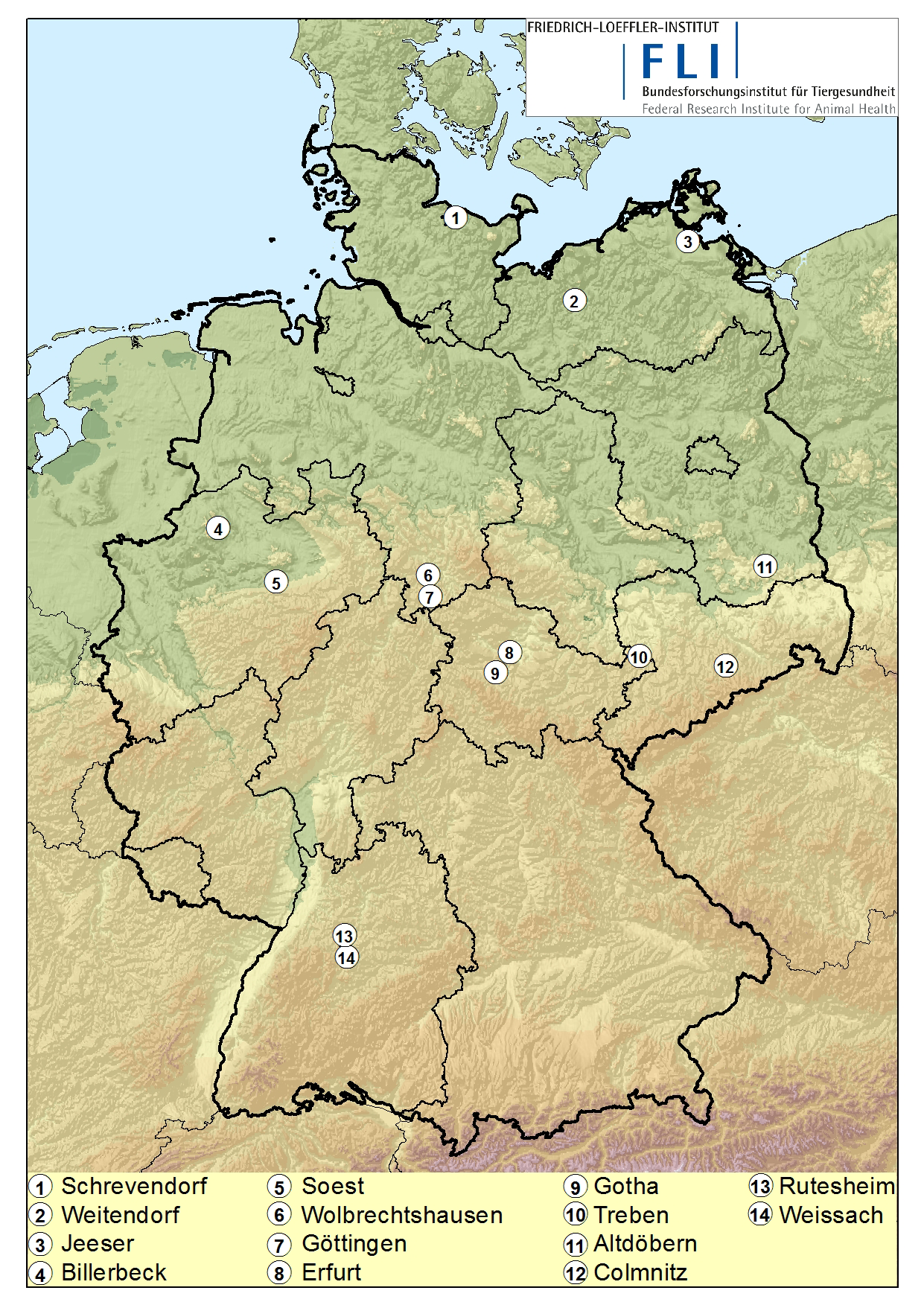

Supplement: S2 Fig — The map was generated using the ArcGis program package. Source: Geobasis-DE/BKG/GeoNutzV. (TIF) [file pone.0140916.s002.tif]
