# Supplementary material for: Identification of Two Novel Members of the Tentative Genus Wukipolyomavirus in Wild Rodents
Source: PLoS One. 2015 Oct 16;10(10):e0140916. doi: 10.1371/journal.pone.0140916 (PMC4608572; doi:10.1371/journal.pone.0140916)
Supplement: S1 Table — (PDF) [file pone.0140916.s003.pdf]

S1 Table. Primers used to detect, amplify and characterize BVPyV and CVPyV genomes.

Primer designation	Sequence (5’-3’)
**Diagnostic PCR primers**	
BVPyV -T-R3	CTGGAAGGTCTCATAAGTTAAAG
BVPyV -T-D3	CTGGCGAAACTGCTAGACAG
CVPyV -T-D4	CTTCTGGAGGCTTCTGCTTTAC
CVPyV -T-R4	CATAGTTCAGGGGGAATCCG
**Genome amplification primers**	
BVPyV -D1	CTTTCCCCCTAACAAATCAAGAAG
BVPyV -R1	CTTAATATCAATGTTCCAGCTGACAAG
CVPyV -D1	GTTGTTTTTGAGGATGTGAAGGGAC
CVPyV -R1	CATAAATTGGTCTATTGCAACACCTAG
**Sequencing primers**	
BVPyV -Tag-R1	GAGTTGCTTGAGAGTTGGGCATTG
BVPyV -Tag-R2	GCTTCGGATCTGCTCAGGGTTC
BVPyV -VP1-R1	CACCAGTGAGACCAAGCATGTCTG
BVPyV -VP2-R1	GCTACCAAAGTTACTAATTCCTC
CVPyV -VP1-R1	GTATAGGGATTCCTTATAGTTCTC
CVPyV -VP1-R2	CAATTCCGCACACCATCATCACTG
CVPyV -VP2-R1	GCTGCACAGTTGGTATGTATGACAAG
CVPyV -Tag-R1	GAATCAGCATTAAAGTGCCTGCTTGC
CVPyV -Tag-R2	GCATCCCATGCATTGCACAGCCTG
**VP1 gene amplification primers**	
BVPyV -ATG	CGTCTAGATGCGCCGGTCGAGAGCACCTTCG
BVPyV -Stop	CGTCTAGTTATGTGTTGAATAAGGGCTGAG
CVPyV -ATG	GCTCTAGAATGCGCCGGTCGAGAGCACCGTCG
CVPyV -Stop	GCTCTAGATTATGTATTAAAGAGGGGCTGGG
**Polyoma nested PCR primers** [a]	
T-1f	GATGTTTCCTTTCTARRTTNAC
T-1r	GCAAAGATCAAAAAAGCATHTGYCA
T-2f	AAATGATCTCTCAAGTTATCNARRTT
T-2r	AAAGGTCCAGTTAATAGTGGNAARAC

[a]Johne R, Enderlein D, Nieper H, Müller H. Novel polyomavirus detected in the feces of a chimpanzee by nested broad-spectrum PCR. *J Virol*, 2005;*79*(6):3883-3887.
