# Supplementary material for: Identification of Two Novel Members of the Tentative Genus Wukipolyomavirus in Wild Rodents
Source: PLoS One. 2015 Oct 16;10(10):e0140916. doi: 10.1371/journal.pone.0140916 (PMC4608572; doi:10.1371/journal.pone.0140916)
Supplement: S2 Table — (PDF) [file pone.0140916.s004.pdf]

S2 Table. Vole trapping site information and genome characterization of BVPyV and CVPyV.

	BVPyV					CVPyV	
Sample number	KS/14/281	KS/14/289	KS/14/328	KS/14/336	KS/13/999	KS/13/947	KS/13/980
GenBank acc. no.	KR612368	KR612369	KR612370	KR612371	KR612372	KR612373	KR612374
Trapping site	Crailsheim, Baden-Wuerttemberg	Crailsheim, Baden-Wuerttemberg	Crailsheim, Baden-Wuerttemberg	Crailsheim, Baden-Wuerttemberg	Gotha, Thuringia	Jeeser, Mecklenburg-Western Pomerania	Gotha, Thuringia
Trapping date	15.08.2013	01.10.2013	14.08.2013	15.08.2013	30.09.2012	04.10.2013	22.09.2012
**Genome length**	**5032 bp**	**5032 bp**	**5032 bp**	**5031 bp**	**5011 bp**	**5024 bp**	**5024 bp**
VP1 ORF length, (genome positions)	1062 bp (1085-2146)	1062 bp (1085-2146)	1062 bp (1085-2146)	1062 bp (1085-2146)	1062 bp (1085-2146)	1065 bp (1081-2145)	1065 bp (1081-2145)
VP1 protein length, aa	353	353	353	353	353	354	354
VP2 ORF length, (genome positions)	1059 bp (57-1115)	1059 bp (57-1115)	1059 bp (57-1115)	1059 bp (57-1115)	1059 bp (57-1115)	1155 bp (57-1211)	1155 bp (57-1211)
VP2 protein length, aa	352	352	352	352	352	384	384
VP3 ORF length, (genome positions)	717 bp (399-1115)	717 bp (399-1115)	717 bp (399-1115)	717 bp (399-1115)	717 bp (399-1115)	816 bp (396-1211)	816 bp (396-1211)
VP3 protein length, aa	238	238	238	238	238	271	271
STag ORF length, (genome positions	648 bp 3927-4574	648 bp 3927-4574	648 bp 3927-4574	648 bp 3926-4573	648 bp 3928-4575	570bp 4005-4574)	570bp 4005-4574
STag length, aa	215	215	215	215	215	189	189
LTag ORF length, (genome positions)	1956 bp (2192-3686, 3792-4001, 4324-4574)	1956 bp (2192-3686, 3792-4001, 4324-4574)	1956 bp (2192-3686, 3792-4001, 4324-4574)	1956 bp (2192-3686, 3791-4000, 4323-4573)	1953 bp (2196-3687, 3793-4002, 4325-4575)	1929 bp (2208-3831, 3949-4002, 4324-4574)	1929 bp (2208-3831, 3949-4002, 4324-4574)
LTag Donor site 1	**caggtacac**	**caggtacac**	**caggtacac**	**caggtacac**	**caggtacac**	**caggtacac**	**caggtacac**
LTag Acceptor site 1	**tttgttttttaggt**	**tttgttttttaggt**	**tttgttttttaggt**	**tttgttttttaggt**	**tttgttttttaggt**	**tctttttttttaggt**	**tctttttttttaggt**
LTag Donor site 2	**atgggaaga**	**atgggaaga**	**atgggaaga**	**atgggaaga**	**atgggaaga**	**atgggaaga**	**atgggaaga**
LTag Acceptor site 2	**ctccctttccagac**	**ctccctttccagac**	**ctccctttccagac**	**ctccctttccagac**	**ctccctttccagac**	**tattattcacagga**	**tattattcacagga**
LTag length, aa	651	651	651	651	650	642	642
X-ORF	4835-4996	4835-4996	4835-4996	4834-4995	4889-66	4534-4959	4534-4959
Putative protein length, aa	53	53	53	53	62	141	141
